# Targeted Development of an Optimised Formulation for 3D-Printing of a Sertraline Hydrochloride-Containing Drug Delivery System with Immediate-Release Characteristics Utilising a Mixture Design

**DOI:** 10.3390/pharmaceutics17091137

**Published:** 2025-08-30

**Authors:** Mirco Bienhaus, Leif Neumann, Charlotte Müller, Frank E. Runkel

**Affiliations:** 1 Department of Life Science Engineering, Institute of Bioprocess Engineering and Pharmaceutical Technology, Technische Hochschule Mittelhessen—University of Applied Sciences, Wiesenstrasse 14, 35390 Giessen, Germanycharlotte.mueller@lse.thm.de (C.M.); frank.runkel@lse.thm.de (F.E.R.); 2 Department of Biology and Chemistry, Justus Liebig University, Ludwigstrasse 23, 35390 Giessen, Germany; 3 Department of Pharmaceutics and Biopharmaceutics, Philipps University Marburg, Robert-Koch-Strasse 4, 35037 Marburg, Germany

**Keywords:** 3D printing, personalised medicine, sertraline hydrochloride, hot-melt extrusion, drug delivery system, immediate release, design of experiment, mixture design

## Abstract

**Objectives:** Although 3D-printing has been identified as a promising technique for personalised medicine manufacturing, developing complex formulations that are suitable for the process can be challenging. This study evaluates the use of a mixture design for the targeted development of an optimised formulation designed for the 3D-printing of oral dosage forms containing the drug sertraline hydrochloride featuring immediate-release drug dissolution. **Methods:** The polymers Eudragit E PO, Kollidon 17 PF and hydroxypropyl cellulose were compared in simple screening experiments regarding their extrudability, printability and disintegration. A combination of Eudragit E PO and Kollidon 17 PF proved superior and therefore served as the basis for the mixture design. The resulting blends were processed via hot melt extrusion to produce filaments, which were then measured for bending stress using a 3-point-bending-test, and 3D-printed sample plates were used to determine the crystallinity index of sertraline hydrochloride using X-ray diffraction in a previously identified range with low interference from the other components. The formulation was optimised using statistically based models with the aim of minimising the bending stress to obtain flexible, process-robust filaments and simultaneously minimising the crystallinity index with the intention of improving the solubility of the drug by maximising its amorphous content. **Results:** The filaments made from the optimised formulation could be reliably printed, and the amorphous state of the active ingredient therein was confirmed. The oral dosage forms produced from these showed immediate release characteristics in an acidic medium. **Conclusions:** This study demonstrates the advantages of a mixture design for optimising complex formulations in a time- and resource-efficient way and could serve as a basis for other research groups to develop innovative, customisable drug delivery systems more effectively.

## 1. Introduction

Oral dosage forms remain one of the most important forms of drug administration, with an estimated market share of around 90% [1] and are widely accepted by patients due to their ease of use [2]. The majority of standardised medications have fixed dosages based on a ‘one size fits all’ approach [3,4]. However, this concept is subject to increasing controversy, partially due to studies suggesting that up to 80% of adverse side effects may be attributed to incorrect dosages [5]. Consequently, the personalisation of medication is increasingly acknowledged as a more advanced therapeutic approach [6].

While established large-scale industrial processes are capable of producing pharmaceuticals with standard dosages cost-effectively and in large quantities, it remains challenging to manufacture individualised dosage forms via this route [3]. Additive manufacturing, also known as 3D-printing, is being recognised as a promising technique for the manufacturing of personalised medicines due to its capability of producing small batches with a high degree of individualisation in terms of shape and dosage [7].

Of the 3D-printing processes used in the pharmaceutical context, fused deposition modelling (FDM) is considered to be one of the most important techniques [8,9]. The filaments used as the printing material can be produced by hot melt extrusion (HME) from thermoplastic polymers serving as a carrier material, often combined with other additives, such as plasticisers and fillers, and the desired active ingredient [10].

Besides the production of filaments for 3D-printing, HME can be used to improve the solubility of poorly soluble drugs [11], which currently make up the majority of all available drugs [12]. The commonly used strategy to achieve this is to convert the crystalline active ingredient into the amorphous state, which increases entropy, enthalpy and free energy, leading to an increase in solubility [13]. The resulting extrudate can then be pelletised and processed conventionally or used as a filament for additive manufacturing [1], although FDM 3D-printing imposes additional requirements on the mechanical properties of the filaments [6].

Many polymers with the necessary quality characteristics, such as biological safety and thermal processability, have already been used to manufacture drug dosage forms with different release profiles, demonstrating the potential of additive manufacturing for the production of personalised medication [14]. Using more complex formulations consisting of several different components enables finer adjustment of the material properties but can be a complex challenge [15,16]. The characterisation of these composite materials using the one-factor-at-a-time (OFAT) approach is time-consuming and resource-intensive. Consequently, alternative methods like the Design of Experiments (DoE) are beneficial in minimising the required experimental effort while enabling predictions within predefined limits and developing the design space [17].

The mixture design applied in this study is a specific type of DoE method for optimising the composition of a formulation based on target parameters, known as responses [18,19]. In accordance with the Food and Drug Administration FDA’s recommended ‘Quality by Design’ (QbD) approach [20], our goal was to systematically develop and optimise a 3D-printable — and therefore customisable — drug dosage form with immediate-release properties since up to 70% of all orally administered pharmaceuticals exhibit this release behaviour [21].

We selected the polymers Eudragit E PO, Kollidon 17 PF and hydroxypropyl cellulose (HPC), as they have already been used in the preparation of other immediate-release dosage forms [14,22]. Simple screening experiments were then carried out to evaluate their extrudability, printability and dissolution behaviour in an acidic medium. A combination of Eudragit E PO and Kollidon 17 PF with stearic acid acting as a plasticiser was found to be superior and therefore formed the basis for our mixture design. Measuring the response values from the DoE runs allowed us to create statistically based models for predicting the response within the possible limits of the component proportions with the aim of optimising the mixture.

Sertraline hydrochloride (sertraline HCl) was selected as a model drug to serve as an example of a pharmaceutical ingredient with potential for 3D-printed personalised dosage forms and to illustrate our proposed DoE-based formulation development process. It is an antidepressant from the group of selective serotonin reuptake inhibitors [23] and, like numerous other drugs, it belongs to the Biopharmaceutical Classification System (BCS) class II, characterised by good permeability but poor solubility [24]. Regarding formulation optimisation, we aimed to minimise the crystallinity index of the active ingredient to improve the solubility by achieving the highest possible proportion of amorphous sertraline HCl—a strategy that is supported by the findings of other research groups who have used HME with different drugs to achieve similar results [11]. We analysed custom-made printed sample plates using X-ray diffraction (XRD) to determine the crystallinity index of the active ingredient within a range that we identified as free of interference from other crystalline peaks.

Antidepressants require careful monitoring of dosage and can cause significant side effects if dosed incorrectly, potentially leading to discontinuation of treatment and relapse into depression, making this group of drugs ideal for personalised therapy [25,26]. We therefore wanted to investigate 3D-printing as a promising approach for manufacturing these personalised pharmaceuticals, especially since, to our knowledge, no printed dosage forms containing sertraline hydrochloride have been produced to date. To ensure reliable printability a filament with suitable mechanical properties is required, so we chose the minimisation of bending stress, which was investigated using a 3-point bending test, as the second optimisation criterion.

After verifying the predictive accuracy of the models through verification runs, also representing the optimised formulation, the filaments could be reliably printed into oral dosage forms and examined for cumulative drug release in an acidic medium. The desired immediate-release characteristic of the oral dosage form was confirmed, and XRD analyses indicated that the active ingredient was present in an amorphous state.

## 2. Materials and Methods

### 2.1. Materials

We used the polymers Eudragit E PO, kindly provided by Evonik Operations GmbH (Darmstadt, Germany) and Kollidon 17 PF, donated by BASF (Ludwigshafen, Germany) as the main components, and stearic acid, purchased from Merck (Darmstadt, Germany), as the plasticiser. Hydroxypropyl cellulose (HPC) was kindly donated by Nisso Chemical Europe GmbH (Düsseldorf, Germany). The active ingredient sertraline hydrochloride was purchased from AK Scientific (Union City, CA, USA). For HPLC analysis, we used methanol (HPLC quality, isocratic grade, VWR international GmbH, Darmstadt, Germany) and acetate buffer (pH 4.5), prepared in-house from sodium acetate (Ph. Eur. grade, Carl Roth GmbH, Karlsruhe, Germany) and acetic acid (VWR international GmbH, Darmstadt, Germany). The hydrochloric acid used for dissolution was bought from Carl Roth GmbH (Karlsruhe, Germany).

### 2.2. Screening of Formulation Excipients

To check for suitable polymers and thereafter to define the DoE limits, the polymers hydroxypropylcellulose (HPC), Eudragit E PO and Kollidon 17 PF were first extruded separately, followed by a mixture of Eudragit E PO and Kollidon 17 PF. The concentration of our chosen plasticiser, stearic acid, was 5% *w*/*w* for each powder mixture, based on our experience and previous literature research [7]. The resulting filaments were analysed for their extrudability, printability and the printed caplets for their weight loss after 30 min in an ZT 221 disintegration tester (ERWEKA GmbH, Langen, Germany) with an acidic media (0.1 M HCl) to simulate gastric conditions [27].

### 2.3. Thermogravimetric Analysis

To ensure that the filament components do not degrade during extrusion, a thermogravimetric analysis was carried out on a TG 209 F1 Iris system (NETZSCH-Gerätebau GmbH, Selb, Germany). For this purpose, 10 mg of each component was weighed into an open aluminium oxide pan (Mettler Toledo GmbH, Gießen, Germany) and measured at a nitrogen flow rate of 20 mL/min. The temperature range analysed was 30 °C to 400 °C at a heating rate of 10 K/min. The data obtained were analysed using NETZSCH Proteus v6.1.0 (NETZSCH-Gerätebau GmbH, Selb, Germany) and plotted via OriginPro v2025 (OriginLab Corporation, Northampton, MA, USA).

### 2.4. Filament Production via Hot Melt Extrusion (HME)

Prior to hot melt extrusion, the powder mixtures were weighed according to the scheme given in Table 1 and Table 5 and then mixed in a simple container mixer driven by an Erweka AR 402 multipurpose drive (Erweka GmbH, Langen, Germany) at 35 rpm for 40 min. Each mixture contained a total mass of 100 g and was subsequently transferred to a ZD 12 FB-C-1M-200/100 gravimetric dosage unit (Three-Tec GmbH, Seon, Switzerland), which was operated at 12 rpm to ensure a constant powder flow for extrusion. Hot melt extrusion was carried out using the ZE 12 mm co-rotating twin-screw extruder (Three-Tec GmbH, Seon, Switzerland) to obtain the filaments from the blends. The barrel of the extruder features 6 separate heating zones, which were adjusted separately during the extrusion process of our screening filaments so that continuous processing of each formulation was assured. The temperature was kept constant at 170 °C for all extrusions of the mixture design. However, the temperature of the heat zone prior to the powder feed was always kept 20 °C lower than the extrusion temperature to prevent clumping and, therefore, clogging of the feed. The molten material was passed through a 2.85 mm nozzle and then cooled on a conveyor belt (Three-Tec GmbH, Seon, Switzerland). To control the filament diameter, an IG-028-CCD-laser-micrometer (Keyence Deutschland GmbH, Neu-Isenburg, Germany) was integrated into the production line and the speed of the conveyor belt was then manually adjusted to ensure the most consistent filament diameter possible (max. tolerated deviation ± 0.05 mm). The finished filaments were stored in a resealable plastic bag to prevent moisture absorption.

### 2.5. 3D Printing of Oral Dosage Forms

**Figure 1:**
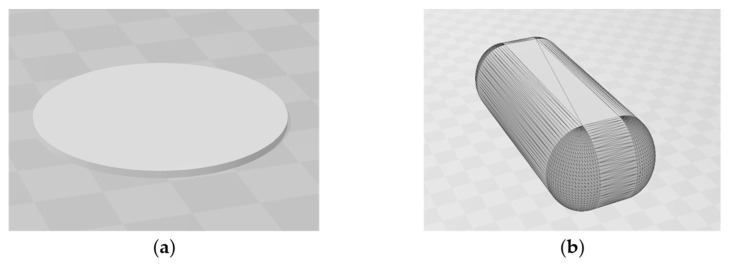
Computer-aided design (CAD) models that served as the template for 3D-printing. (**a**) Flat, cylindrical form (40 mm diameter), suitable for direct measurement in XRD defined as “sample plate”; (**b**) oblong, fully filled form (12 mm length, 5 mm width, 4 mm height) named “caplet”.

The oral dosage forms were made via the fused deposition modelling technique, for which the Ultimaker S5 3D-printer (Ultimaker B.V., Utrecht, The Netherlands) was utilised. The print head was equipped with a BB 0.8 mm nozzle and is capable of processing filaments with a diameter of 2.85 mm. The computer models used as templates for the printing process were created using the open-source tool Autodesk^®^ Tinkercad^®^ (Autodesk Inc., San Rafael, CA, USA) and are visualised in Figure 1. Based on this, the paper will refer to the terms “sample plate” and “caplet” for these two different types. We designed the shape of the caplet based on the observation that patients generally prefer capsule-like dosage forms with strongly curved arching shapes for medication and that oblong shapes were preferred if larger dosages were required [28].

To convert the CAD files into commands that the printer can process (slicing), we used the open-source software Cura (v5.2 and newer, Ultimaker B.V., Utrecht, The Netherlands), which also allowed us to further adjust the printing settings to improve the print quality. The different filaments required a precise adjustment of the printer settings, so that only a printing speed of 10 mm/s for the first layer and 20 mm/s for all layers based on it, as well as a layer height of 0.2 mm for the first layer and 0.1 mm for all layers on top of it were identical for all prints. The bed temperature, as well as material flow and printing temperatures, were adjusted for each blend to produce a high-quality print, as shown in Table 2.

### 2.6. Design of Experiment (DoE)

To investigate the effects of the filament composition, we configured a l-optimal mixture design, which is commonly used for optimisation studies [18], utilising Design Expert software (Version 23.1.8). This type of design is characterised by the condition that the sum of all components must always equal 100% [29]. These components were limited in their minima and maxima, according to Table 3, while keeping the sertraline HCl concentration fixed at 10% *w*/*w* in each formulation [30]. A drug loading of 10% *w*/*w* corresponds to a quantity of approximately 25 mg of sertraline HCl using our specified caplet size, which may represent a typical starting dose for initial therapy, depending on the patient.

Based on our literature research and previous experience, we defined the bending stress and crystallinity index of the active ingredient as the critical response parameters [31,32]. Our goal was to minimise both parameters to produce a filament that is as amorphous and flexible as possible, making it more suitable for 3D-printing. The test plan we generated consisted of 13 runs, of which 4 were replicates for error calculation and 3 were used to determine the lack of fit. All trials were randomised to minimise the influence of systemic errors.

### 2.7. Three-Point-Bending-Test

A three-point bend test was carried out to analyse the mechanical properties of the DoE formulations. For each formulation, 5 filament sections were randomly selected and cut into 50 mm segments. The measurement was performed in a texture analyser (Hegewald and Peschke Mess- und Prüftechnik GmbH, Nossen, Germany) which was equipped with a three-point bend equipment. The distance between the support pins was 35 mm, and the test speed was set to 100 mm/min. The data were collected using the LabMaster software (version 2.3.5.9 and newer) and then analysed using OriginPro v2025 (OriginLab Corporation, Northampton, MA, USA). The measured parameters were the applied force (N) and the deflection (mm). As it was assumed from previous tests that not all formulations would exhibit complete breakage of the filaments, the bending stress $\sigma_{f}$ at 1% flexural strain $\varepsilon_{f}$ was calculated using Equations (1) and (2) in order to also enable soft filaments to be measured and compared [33].

(1) $$\varepsilon_{f}=\frac{600\cdot s\cdot d}{L^{2}}$$

where

$\varepsilon_{f}$ is the flexural strain in %;

*s* is the deflection in mm;

*d* is the sample thickness in mm;

*L* is the distance between the supports in mm.

(2) $$\sigma_{f}=\frac{8*F*L}{\pi *d^{3}}$$

where

$\sigma_{f}$ is the stress in N·mm^−2^;

*F* is the force in N;

*L* is the distance between the supports in mm;

*d* is the sample thickness in mm.

### 2.8. X-Ray Diffraction

XRD measurements were carried out to identify the solid-state of the raw materials and the printed sample plates. The analysis was performed using a D2 Phaser (Bruker Corporation, Billerica, MA, USA) equipped with a copper anode (1.54184 Å) at a current of 10 mA and a voltage of 30 kV. The samples were placed in a zero-background PMMA sample holder and analysed in continuous scanning mode from 5–32° 2θ at a step size of 0.22° 2θ and a step time of 1 s. The obtained data were then processed and plotted using OriginPro v2025 (OriginLab Corporation, Northampton, MA, USA). The crystallinity index CI was calculated using the following equation [34]:

(3) $$CI=\;\frac{A_{cr}}{A_{sample}}$$

where

*CI* is the crystallinity index;

$A_{cr}$ is the area of the crystalline peaks;

$A_{sample}$ is the area of the sample.

### 2.9. Dissolution Studies

Following the printing of the dosage forms, they were analysed for their active ingredient release. Based on the European Pharmacopoeia requirements, an Apparatus 2 structure was selected for this purpose. Using a 708-DS dissolution apparatus (Agilent Technologies Deutschland GmbH, Waldbronn, Germany), 200 mL of 0.1 M hydrochloric acid per vessel was heated to 37 ± 0.5 °C, and the paddle rotation speed was set to 100 rpm. For each measurement, three caplets containing the active ingredient were analysed alongside one caplet without the active ingredient. Additionally, a sertraline-containing caplet that we produced with hydroxypropyl cellulose (HPC) instead of Eudragit E PO and Kollidon 17 PF as the main polymer was selected as a reference formulation for an unoptimised 3D-printed dosage form with expected rapid drug release [22,35,36]. At fixed time points of 0, 2, 6, 10, 15, 30, 45, 60, and 120 min, an 850-DS dissolution sampling station (Agilent Technologies Deutschland GmbH, Waldbronn, Germany) removed 1.5 mL of the respective sample and replaced it with the same amount of fresh medium. The samples were then centrifuged, diluted with 0.1 M sodium hydroxide solution and methanol in a ratio of 1:1:8, and analysed for their active ingredient content using HPLC with the method described hereafter. The dissolution studies were performed in triplicate.

### 2.10. High Performance Liquid Chromatography (HPLC) Analysis

The drug content of the samples was determined by HPLC. The system (LaChrom Elite system, Hitachi, Tokyo, Japan) consisted of an HPLC pump (Hitachi LaChrom Elite L-2130), an autosampler (Hitachi LaChrom Elite L-2200) and a DAD detector (Hitachi LaChrom Elite L-2455). The column utilised was a LiChrospher^®^ 100 RP-18 end-capped column (4 mm × 125 mm; 5 µm, Merck, Darmstadt, Germany) coupled with a precolumn. Peaks were analysed using EZChrom Elite software (version 3.3.2 SP2, Hitachi, Tokyo, Japan). The eluent consisted of methanol and acetate buffer (10 mM, pH 4.5), which was prepared in-house from sodium acetate and acetic acid and then filtered before being used in the system using a 0.45 µm filter. The measurements were carried out under isocratic conditions (90% methanol and 10% buffer *v*/*v*) with a flow rate of 1.5 mL/min, a temperature of 50 °C and an injection volume of 50 µL. The UV absorbance of the samples was measured at 215 nm.

## 3. Results

### 3.1. Screening Observations for the Selection of DoE Limits

When selecting suitable polymers for pharmaceutical use in HME coupled with additive manufacturing, extrudability and printability are typically examined as critical parameters [14,37]. We defined extrudability as the capability to produce filaments with a diameter of 2.85 ± 0.1 mm, which was a requirement for the 3D-printer being used. Printability was classified as positive if the filaments could be processed into the desired caplet shape. We selected the disintegration time of the printed caplets in 0.1 M HCl as an additional criterion to make preliminary predictions about the dissolution of the formulations in an acidic environment. Conceptionally oriented towards the criteria of the US FDA 2018 guidance for highly soluble compounds, the caplets should have lost at least 80% of their initial mass within the first 30 min [38].

**Figure 2:**
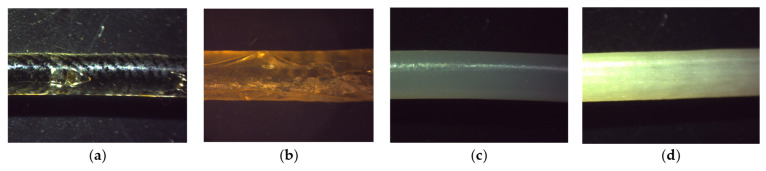
Extruded filaments (diameter of 2.85 ± 0.1 mm) from the screening phase: (**a**) E-S; (**b**) K-S; (**c**) H-S; (**d**) E-K-S.

It was possible to extrude all powder blends continuously over the entire process duration, as shown in Table 4 and Figure 2. However, neither the filaments with Eudragit E PO (E-S) nor those with Kollidon 17 PF (K-S) as the main polymer were suitable for reliable printing. The powder mixture K-S resulted in an extremely brittle and highly fragile filament that was not suitable for mechanical feeding [39]. In contrast, the filament E-S proved to be too soft to be continuously conveyed to the print head by the feeding mechanism, which was also reported by Nasereddin et al. [40] for filaments with a high Eudragit E PO content. We were also able to confirm the observation that soft filaments tended to wind up on the way to the print head and thus clogging it [41].

However, short sections of filament E-S could be inserted into the Bowden tube of the printer and then used to print single caplets with the aid of a flexible PLA filament acting as a piston [31]. As this procedure did not enable continuous production, the printability was still rated as insufficient. HPC has previously been used in other studies to successfully produce precise structures using FDM [35], and this was also accomplished with our formulation H-S. Surprisingly, we found that it was also possible to both extrude and print the composite filament E-K-S with ease.

The obtained caplets were analysed for their disintegration in 0.1 M HCl, which revealed that the fastest disintegration was shown by the caplets of the combined formulation E-K-S, followed by those of the formulation E-S. The caplets containing HPC as their main polymer were not fully disintegrated after 60 min, therefore exceeding our target. Although the disintegration time of the drug-free caplets cannot be directly extrapolated to the final dissolution time of the drug-containing oral dosage forms, this screening experiment allowed us to quickly and cost-efficiently preselect potentially suitable components without the need for complex HPLC analysis at this stage. Since the combination of the polymers Eudragit E PO and Kollidon 17 PF enabled both extrudable and printable filaments and the resulting caplets fulfilled our disintegration criterion, they were used to create the mixture DoE.

### 3.2. Selecting the Extrusion Temperature

**Figure 3:**
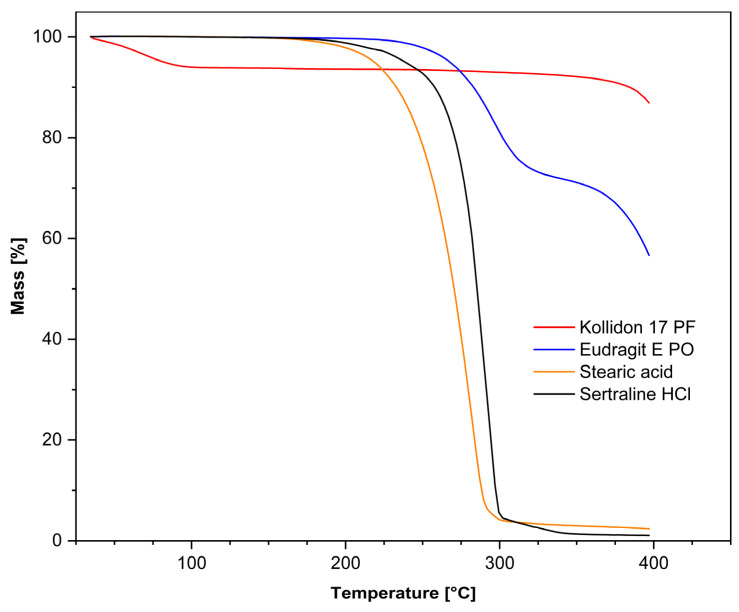
Thermal analysis of the filament components for the DoE formulations. Each sample (10 mg) was analysed using the same TG 209 F1 Iris system at a nitrogen flow rate of 20 mL/min over a temperature range of 30 °C to 400 °C with a heating rate of 10 K/min.

To determine the final extrusion temperature for our mixture design, we first measured the degradation temperatures of the raw materials. The highest possible temperature without visible degradation of the component was defined as the maximum temperature for extrusion. The TGA measurements of the polymers (Figure 3) showed that Eudragit E PO only began to lose mass from 234 °C onwards, whereas Kollidon 17 PF showed the earliest mass loss (~5%) up to a temperature of 110 °C, which we attributed to water loss due to the hygroscopic nature of Kollidon 17 PF [21,42]. After that, no mass loss was seen until about 200 °C. This is consistent with the results of Alshahrani et al. [43], whose formulations with Kollidon 17 PF were shown to be thermally stable up to over 220 °C. However, since the plasticiser we used, stearic acid, showed a rapid loss of mass from 180 °C onwards, we saw this as the limiting factor for our extrusion temperature. Sertraline HCl appeared to be stable up to 196 °C. Conversely, the research group led by Zayed et al. [44] recorded the onset of degradation beginning at 180 °C, so that the extrusion temperature was set at 170 °C for all DoE formulations as a precaution and in accordance with the decomposition temperature of the plasticiser.

### 3.3. Setting up the Mixture Design and Sample Preparation

Based on the formulation E-K-S from our screening phase, we determined the constraints of the mixture design (Table 3). As formulation E-S was not suitable for continuous printing in our screening tests but could be processed in general, Eudragit E PO was chosen as the main component at a concentration of 40–75% *w*/*w*. Kollidon 17 PF, which was not printable in high concentrations in the preliminary tests, contributed between 10% and 40% *w*/*w* to the powder mixture and the plasticiser was only used at 5–10% *w*/*w*, as preliminary tests with a higher stearic acid content led to excessively soft and unfeedable filaments. The proportion of sertraline HCl was kept constant at 10% *w*/*w*, as we wanted to focus on the effects of the excipients on the response parameters. However, we would like to point out that adding the active ingredient to the mixture can have a significant impact on the mechanical properties. While the effect of different sertraline loadings was not the subject of our study, to avoid affecting the final amount of active ingredient in the caplets, we observed that the sertraline-containing filaments appeared more brittle than the drug-free filaments from the screening tests. When other research groups change the drug loading or use a different active ingredient, an additional influence on the mechanical properties can be expected.

**Figure 4:**
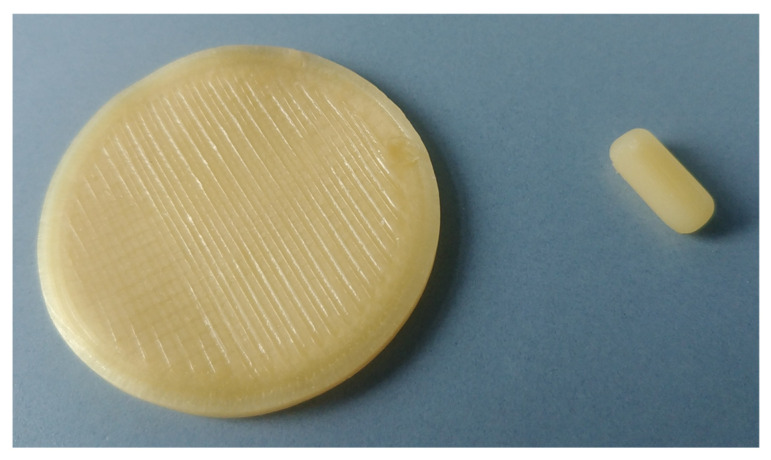
Example of a sample plate produced for XRD analysis (40 mm diameter) alongside the printed caplet used for drug dissolution testing (12 mm length, 5 mm width, 4 mm height).

All 13 formulations (Table 5) could be extruded and printed. While the caplets were generally easy to remove from the build plate of the 3D-printer, the sample plates for XRD measurements strongly adhered due to their large contact surface area and had to be carefully removed to prevent deformation. However, attaching aluminium foil to the printing surface before starting the process minimised this problem. The caplets and sample plates were amber-coloured and showed no optical inhomogeneities, as shown in Figure 4.

### 3.4. Effect of the Mixture Composition on the Bending Stress

Different models (linear, quadratic, cubic and special cubic) were generated using the observed response data, for comparison and selection of the best fitting one. We chose the model with a significant *p*-value (*p* < 0.05) and the highest non-significant lack-of-fit (*p* > 0.05, as we want the selected model to fit).

**Figure 5:**
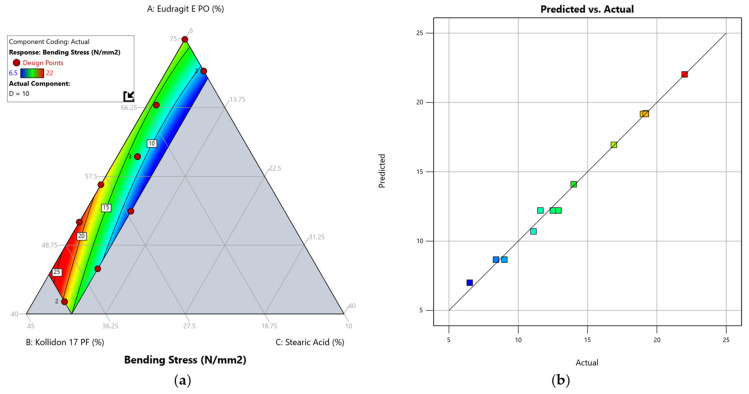
Model graphs of the quadratic model for the response bending stress: (**a**) contour plot visualising the effect of the component ratio on the predicted response. (**b**) Predicted vs. actual plot to evaluate the correlation between experimental and predicted outcomes. The design points are represented by red dots alongside the number of replicates where applicable. For better visualisation, the response value is highlighted via colour grading (blue = low value, red = high value) according to the legend in the top left corner.

We identified that a quadratic model best describes the effect of our mixture composition on the response bending stress. The summary of the analysis of variance (ANOVA) results (Table 6) shows that the model is significant with an F-value of 253.96 and that there is only a 0.01% chance that such a high F-value can occur due to noise. The lack-of-fit is insignificant, as reflected by a *p*-value of 0.6683. Furthermore, we compared the adjusted regression coefficient (R = 0.9906) to the predicted regression coefficient (R = 0.9839). A difference of less than 0.2 indicates that these are in reasonable agreement and that the values predicted by the model for the bending stress are within a 95% confidence interval (CI) of the measured results [45]. The adequate precision is a measure of the signal-to-noise ratio and indicated an adequate signal with a value of 47.084 (a ratio > 4 is desirable). The actual versus predicted plot of the linear model (Figure 5) shows only small deviations between the predicted response values and the experimental measurements which suggests that the chosen model provides a good predictability [46]. The quadratic model was therefore used to navigate the design space, where the response bending stress (Y_1_) is described by the following formula:

Y_1_ = 0.317735 A + 1.53842 B − 5.40646 C − 0.020071 AB + 0.050577 AC + 0.016941 BC (4)

If a variable has a positive sign in front of it, the response value increases as the variable value rises. A negative sign, inversely, lowers the response value as the variable value rises. As illustrated by the contour plot (Figure 5) and the upper Equation (4), an increase in the plasticiser content (variable C) in the analysed filament compositions had the biggest impact on reducing bending stress. This confirms our selection of stearic acid as an effective plasticiser for the in-house production of filaments and is consistent with the results of Desai et al. [47], who reported a plasticisation effect for stearic acid in binary and tertiary mixtures. We attribute this to the relatively small molecular size of stearic acid (284.48 g/mol), which is a common characteristic of plasticisers in polymer melts in general and may increase the overall molecular mobility by becoming embedded between the polymer chains [48].

The Kollidon 17 PF content (variable B) had a remarkably stronger effect on bending stress than the amount of Eudragit E PO (variable A). This behaviour was also demonstrated by our experimentally collected data, as the highest measured value for bending stress was found for DoE-run R10, a formulation with the lowest possible plasticiser content (5% *w*/*w*) and a high Kollidon 17 PF content (33.31% *w*/*w*). Accordingly, the lowest measured value for bending stress was found for DoE-run R1, the formulation with the highest possible stearic acid content (10% *w*/*w*) and low Kollidon 17 PF content (26.93% *w*/*w*).

### 3.5. Effect of the Mixture Composition on the Crystallinity Index After Printing

As the physical state of the active ingredient can have a considerable influence on its release behaviour [49], we investigated this using XRD. While the powdered starting materials could be measured directly with the instrument, this was not possible for the printed caplets. To overcome that issue, individually designed sample plates were printed from the same filaments.

The variety of published approaches for calculating the crystallinity index, which we use as an indication of the presence of a crystalline active ingredient in the printed sample, made it difficult for us to select a suitable method for collecting the response data. While it has been demonstrated that these methods can produce varying absolute results when compared with each other, the quantification of relative differences between samples was found to be barely affected [50]. We would therefore like to state that the method we have chosen aims to quantify the relative differences within a similar formulation [34] in order to optimise within the design space of a mixture design.

**Figure 6:**
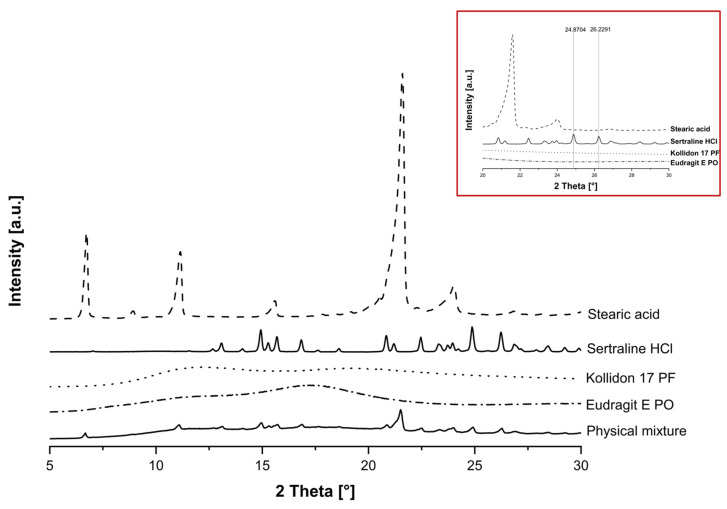
XRD diffractogram of the raw materials and their physical mixture to visualise the interference of the different peaks. The inlet highlights the characteristic peaks (24.87° and 26.23°) of sertraline HCl, which we chose for the relative quantification of the crystallinity index. All samples were analysed with a D2 Phaser (copper anode with 1.54184 Å) at a current of 10 mA and a voltage of 30 kV using the continuous scanning mode from 5 to 32° 2θ at a step size of 0.22° 2θ and a step time of 1 s.

The XRD data for powdered sertraline HCl (Figure 6) showed peaks at 13.10°, 14.90°, 15.68°, 16.80°, 20.86°, 22.47°, 24.87° and 26.23°, many of which are characteristic for the substance [51] and, confirming our expectations, indicate the presence of the crystalline form. However, as there were large overlaps between the sertraline HCl and stearic acid peaks in the 5–24° 2θ range, only the peaks at 24.87° and 26.23° were analysed to calculate the crystallinity index (Figure 6, inlet). The analysis of Eudragit E PO and Kollidon 17 PF revealed no crystalline peaks within the scan range examined, which reflects the expected characteristic of mostly typical amorphous polymers.

**Figure 7:**
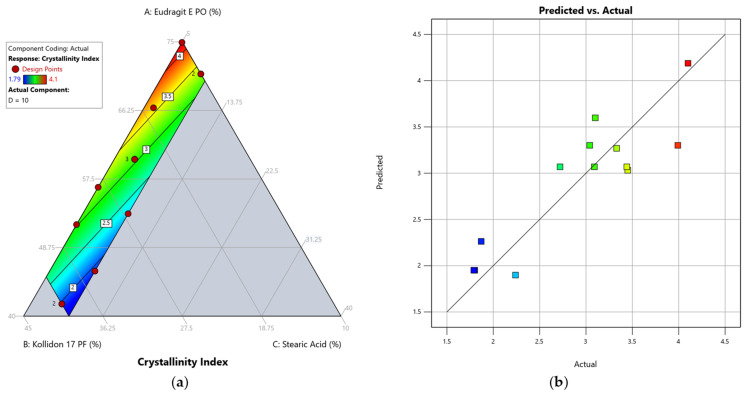
Model graphs of the linear model for the response crystallinity index: (**a**) contour plot visualising the effect of the component ratio on the predicted response. (**b**) Predicted vs. actual plot to evaluate the correlation between experimental and predicted outcomes. The design points are represented by red dots alongside the number of replicas where applicable. For better visualisation, the response value is highlighted via colour grading (blue = low value, red = high value) according to the legend in the top left corner.

The relationship between the composition of the formulation and the response crystallinity index was best described by a linear model. The F-value of 18.97 indicated a significant model, with only a 0.04% chance that such a high F-value could occur due to noise (Table 7). The lack-of-fit is insignificant with a *p*-value of 0.6190 and the adjusted regression coefficient is in reasonable agreement with the predicted regression coefficient with a difference of less than 0.2. Adequate precision was indicated by a value of 12.0762, meaning the linear model could be used to navigate the design space. The actual versus predicted plot of the linear model (Figure 7) shows greater deviation between the predicted response values and the experimental measurements compared to the quadratic model of the bending stress, reflecting the smaller correlation coefficient graphically. However, since the ANOVA did not reveal any exclusion criteria, the model was retained to determine experimentally whether the model can be confirmed through validation runs. The formula for calculating the response crystallinity index (Y_2_) for our formulation is given below:

Y_2_ = 6.41737 A + 1.44073 B − 15.30758 C (5)

As illustrated in the contour plot (Figure 7) and the Equation (5) above, we found that the stearic acid content (variable C) had the greatest positive influence on reducing the crystallinity index in the formulations. Both polymers had a negative influence on the reduction of the crystallinity index, but the strength of their influence differed considerably. The influence of Kollidon 17 PF (variable B) was four times weaker, meaning an increased Kollidon 17 PF ratio in relation to Eudragit E PO (variable A) was found to be more effective in lowering the crystallinity index. This tendency was also observed in our experimental measurements. The formulation DoE-Run 11, which contained the lowest possible amounts of Kollidon 17 PF and stearic acid, exhibited the highest crystallinity index, while DoE-Run 5, which contained the highest possible amount of Kollidon 17 PF and a large amount of plasticiser (8.44% *w*/*w*), showed the lowest crystallinity index.

Kollidon 17 PF is recognised for its use as a solubilising agent and crystallisation inhibitor [14,52], which was previously attributed by Chen et al. [53] to the formation of hydrogen bonds between the carbonyl groups of the pyrrolidone ring of the Kollidon 17 PF molecules and various nitrogen compounds of the drug under investigation. To analyse these and other potential interactions between sertraline HCl and the excipients, additional investigations, for example in the form of infrared spectroscopy, should be carried out in the future.

### 3.6. Optimisation of the Formulation and Confirmation of the Model

Setting up the regression models for the responses allowed us to optimise the formulation within the investigated design space. Our goal was to minimise bending stress to obtain flexible and reliably printable filaments as well as to minimise the crystallinity index to keep the crystalline content of the active ingredient in the printed form as low as possible. Different formulations were suggested based on their desirability, which can range from d = 0 (undesirable) to d = 1 (highly desirable). The formulation with the highest desirability is considered to be the optimal solution [32,54,55].

We ranked the reduction of the crystallinity index higher than that of the bending stress, as we were able to print all the filaments of the DoE runs, despite this sometimes requiring the use of a PLA filament as a piston and we therefore considered the bending stress to be less crucial for the quality of the final product. The underlying criteria for the numerical optimisation process and their respective importance are shown in Table 8.

**Figure 8:**
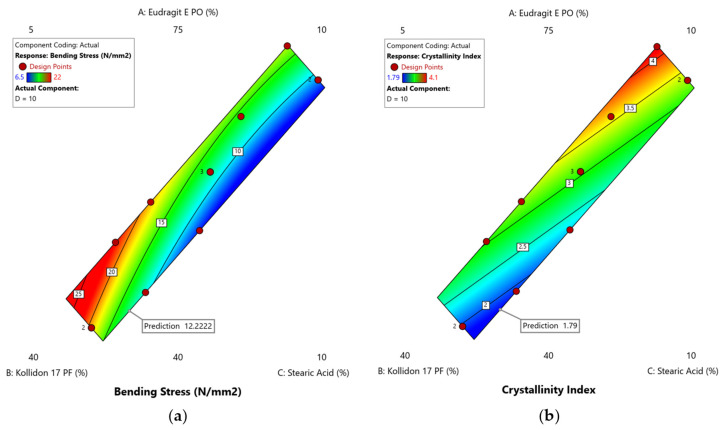
Contour plots illustrating the predicted response for the proposed optimal formulation (marked by the grey-framed flag) in terms of (**a**) the bending stress and (**b**) the crystallinity index. For better visibility only the zoomed-in view is shown. For better visualisation, the response value is highlighted via colour grading (blue = low value, red = high value) according to the legends in the top left corner of each graph.

The suggested formulation with the highest desirability (d = 0.926) consisted of 43.57% Eudragit E PO, 36.43% Kollidon 17 PF, 10.00% stearic acid and 10.00% sertraline HCl (all percentages are *w*/*w*) and is graphically represented by the marker in the contour plots shown in Figure 8. To validate the predicted response values of 12.22 N/mm^2^ for the bending stress and 1.79 for the crystallinity index, three extrusions (from here on referred to as ‘confirmation runs’) were performed with this composition. The experimental values were compared with the predicted values (Table 9) and were found to be within the desired confidence interval (CI) of 95%, which confirmed our models.

### 3.7. Dissolution Studies and Solid State Analysis

To verify whether the optimised formulation demonstrates immediate release properties, dissolution studies were carried out in 0.1 M HCl to mimic gastric conditions. The cumulative drug release should be at least 80% after 30 min to confirm the desired release behaviour [38]. As the printed caplets of formulation H-S from the screening trials lost approximately half their weight during 30 min of disintegration testing, and as drug-containing dosage forms with hydroxypropyl cellulose as the main polymer exhibiting immediate release properties had already been described [22], a modified formulation containing sertraline hydrochloride was prepared as an example of a non-optimised 3D-printed dosage form and was analysed under identical testing conditions. This formulation (from here on referred to as “H-S-S”) contained 85% HPC, 10% sertraline HCl and 5% stearic acid (all percentages are *w*/*w*) and was extruded and printed at the same temperatures.

Both the optimised and the H-S-S formulation were found to be extrudable and easily printable. Each filament was flexible enough to withstand the stresses of the manufacturing process, enabling the printing of the desired caplets. The reduction of the bending stress proved to be an effective way to ensure reliable processability. While other research groups have already identified the measurement of mechanical properties as a useful approach for developing 3D-printed dosage forms [7,40,56,57], our experiments have shown that the bending stress can even be predicted using a mixture design and the corresponding model. This allows the flexible adaptation of the mechanical properties to the requirements of the manufacturing process.

**Figure 9:**
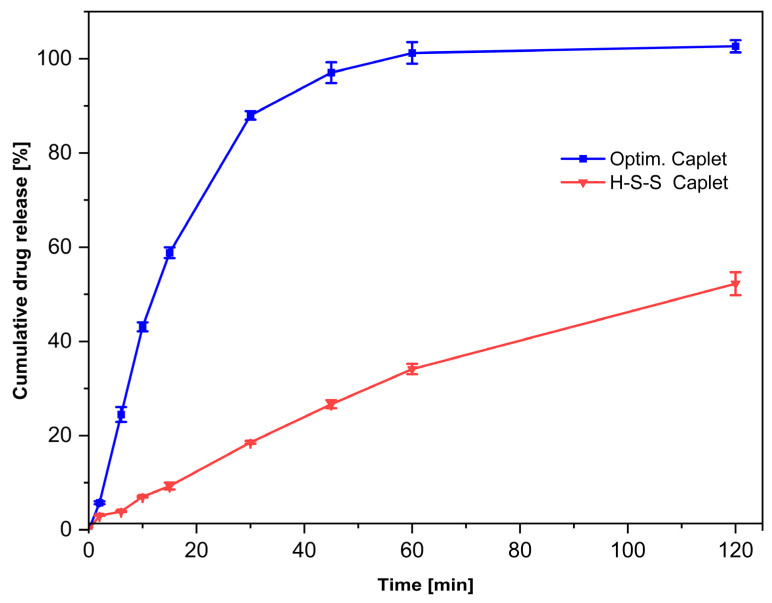
In vitro cumulative drug release of sertraline hydrochloride from the 3D-printed caplets made from the optimised and the H-S-S formulation tested in acidic media for 120 min. (*n* = 3, mean ± SD).

The cumulative drug release of sertraline HCl from the caplets in an acidic medium (0.1 M HCl) was measured and the results are shown in Figure 9. We found that after 30 min, 87.99 ± 0.90% of the drug had been released from the caplets from the optimised formulation, therefore achieving the targeted immediate release behaviour [38]. In contrast, the H-S-S caplets only showed a drug release of 18.26 ± 0.30% after 30 min.

As the amount of the active ingredient, as well as the extrusion and printing parameters, were identical for both formulations, we attribute this difference to the different polymers and varying plasticiser content. Eudragit E PO is a polymer known for its pH-dependent solubility in gastric conditions [14,58], which can be attributed to the protonation of the dimethylamino groups at this pH value [59]. As indicated by our screening experiments, the combination with Kollidon 17 PF also dissolved quickly in the acidic medium, so that after 60 min no more caplets were visible in the vessels. To our surprise, the caplets from formulation H-S-S released the active ingredient even more slowly than expected from the screening trials and even after 60 min only about one-third (34.12 ± 1.12%) of the active ingredient was released.

**Figure 10:**
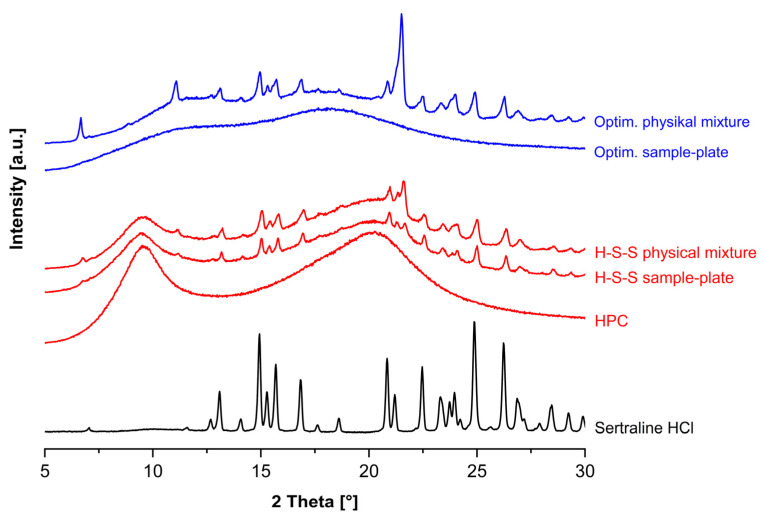
XRD diffractograms of the optimised and the H-S-S formulation (physical mixture and printed sample plate). The powdered sertraline hydrochloride and pure HPC are shown for reference. All samples were analysed with a D2 Phaser (copper anode with 1.54184 Å) at a current of 10 mA and a voltage of 30 kV using the continuous scanning mode from 5 to 32° 2θ at a step size of 0.22° 2θ and a step time of 1 s.

While the fast erosion of the carrier material per se has a substantial impact on drug release from printed carrier systems [31,35], we assume that the different dissolution rates are influenced by additional effects. The XRD analysis of the formulations (Figure 10) revealed that the non-optimised H-S-S composition still showed crystalline peaks in the printed sample plates, whereas these were not observed in the optimised formulation. We therefore conclude that sertraline HCl was successfully converted to the amorphous state in the optimised formulation and that the higher molecular mobility of the active ingredient compared to its crystalline form increased the solubility of the drug [13,60,61].

## 4. Conclusions

This study demonstrates the successful application of a mixture design to optimise a sertraline hydrochloride-containing formulation for extrusion into flexible filaments and subsequent printing into caplets with immediate release characteristics. Simple screening experiments for the evaluation of extrudability, printability and dissolution in acidic media allowed fast pre-selection between the polymers Eudragit E PO, Kollidon 17 PF and HPC to narrow down the most suitable components. While stearic acid was effective in enabling the extrusion of all polymers, only filaments containing HPC or a combination of Eudragit E PO and Kollidon 17 PF could be continuously printed, highlighting the importance of composites in pharmaceutical 3D printing.

The composition of Eudragit E PO, Kollidon 17 PF and stearic acid, with a fixed proportion of 10% *w*/*w* sertraline hydrochloride, was optimised using a mixture design. The aim was to minimise bending stress to obtain flexible filaments and to minimise the crystallinity index for the highest possible proportion of amorphous active ingredient. The three-point bending test and the XRD analysis of printed sample plates proved to be effective methods for creating valid statistical models to predict the target parameters.

To the best of our knowledge, our method for the relative quantification of the crystallinity index of a target substance (e.g., an active ingredient) in a mixture containing several partially interfering components is a novel and promising approach for the optimisation of complex formulations for 3D-printing in the pharmaceutical field. However, as an important step, we recommend that other research groups first complete an XRD analysis of all planned formulation components to estimate the extent of potential peak overlap. If the signals do not overlap, the evaluation should be both simpler and more precise. In contrast, if the signals overlap excessively, it may be necessary to switch to a different response parameter or alternative analysis method.

We would like to emphasise that self-printed samples can be adapted to the analytical setup in a flexible way and the XRD measurements do not destroy the specimens, allowing the analysis of the same samples for stability studies in the future. Further investigations involving new active ingredients and additional analysis techniques (e.g., infrared spectroscopy and differential scanning calorimetry) should be conducted to verify the applicability of this method to different formulations.

The formulation optimised in this study could be successfully 3D-printed continuously and showed faster drug release compared to a non-optimised formulation which we attributed to both the polymer properties and the active ingredient being in an amorphous state. Future studies could adopt our approach to develop and optimise new formulations for 3D-printing with different active ingredients and excipients by using a similar methodology or expand the mixture design to include additional process variables, like differing extrusion temperatures or varying drug loadings. We recommend first defining the critical quality attributes, such as the desired release characteristics (which may lead to a more complex dissolution analysis involving a pH-shift), dosing range and therefore the necessary drug loading of the filaments, as well as the mechanical requirements of the manufacturing process. Based on this, simple screening experiments can speed up the selection from the variety of currently available polymers and additives. We also suggest the combination of several polymers to create fine-tunable composite materials, which can then be optimised systematically using a mixture design to obtain a reliably processable formulation with the desired properties for the manufacturing of the customisable drug dosage form. Before the developed formulation can be used for personalised patient treatment, its printability in other 3D-printers (ideally one developed specifically for pharmaceutical use) should be tested alongside long-term stability tests and extensive clinical studies of the developed oral dosage form.

## Figures and Tables

**Table 1:** Composition of the powder blends of the screening formulations based on their percentage by weight (% *w*/*w*). The names reflect the ingredients contained in each formulation. E = Eudragit E PO, K = Kollidon 17 PF, H = HPC SL, S = Stearic acid.

Blend-Name	Eudragit E PO (% *w*/*w*)	Kollidon 17 PF (% *w*/*w*)	HPC (% *w*/*w*)	Stearic Acid (% *w*/*w*)
E-S	95	-	-	5
K-S	-	95	-	5
H-S	-	-	95	5
E-K-S	65	30	-	5

**Table 2:** Printing parameters for the screening-filaments and DoE-filaments. All settings were configured in the slicing software Cura (version 5.2 and newer) and then submitted to the Ultimaker S5 3D-printer to start the process.

Printing Parameter	E-S	K-S	H-S	E-K-S	DoE-Runs
Bed temperature	80 °C	70 °C	70 °C	70 °C	70 °C
Printing temperature	170 °C	170 °C	160 °C	165 °C	160 °C
Printing speed	20 mm/s	20 mm/s	20 mm/s	20 mm/s	20 mm/s
Layer height	0.8 mm	0.8 mm	0.8 mm	0.8 mm	0.8 mm
Layer width	0.1 mm	0.1 mm	0.1 mm	0.1 mm	0.1 mm

**Table 3:** Constraints of the formulation components of the mixture design demonstrating their overall minimum and maximum possible percentage by weight (% *w*/*w*) in each mixture.

Mixture Component	Proportion of Powder Blend (% *w*/*w*)	
	Low Limit	High Limit
Eudragit E PO	40	75
Kollidon 17	10	40
Stearic acid	5	10
Sertraline HCl	10	10

**Table 4:** Extrusion and printing temperatures of the screening formulations with the results regarding their extrudability, printability and disintegration time in acidic media.

Blend-Name	Extrudability	Extrusion Temperature (°C)	Printability	Printing Temperature (°C)	Disintegration Time (min)
E-S	(+)	120	(−) ^1^	170	19.27 ± 0.75
K-S	(+)	170	(−)	-	-
H-S	(+)	150	(+)	160	>60
E-K-S	(+)	160	(+)	165	28.00 ± 1.63

^1^ could only be printed with the aid of a PLA filament piston.

**Table 5:** Composition of the powder blends of the mixture design runs (R) based on their percentage by weight (% *w*/*w*) and the experimentally determined response values of the bending stress (Y_1_) and the Crystallinity Index (Y_2_).

Blend-Name/ DoE-run (R)	Eudragit E PO (% *w*/*w*)	Kollidon 17 PF (% *w*/*w*)	Stearic Acid (% *w*/*w*)	Sertraline HCl (% *w*/*w*)	Bending Stress (N/mm^2^)	Crystallinity Index
R1	53.07	26.93	10	10	6.54	1.87
R2	70.9	10	9.1	10	9.02	3.99
R3	45.75	34.25	10	10	11.15	2.24
R4	60.01	22.74	7.25	10	12.53	3.44
R5	41.56	40	8.44	10	19.16	1.79
R6	60.01	22.74	7.25	10	12.86	2.72
R7	66.58	17.38	6.04	10	14.03	3.10
R8	56.45	28.55	5	10	19.18	3.33
R9	60.01	22.74	7.25	10	11.63	3.09
R10	51.69	33.31	5	10	21.71	3.45
R11	74.93	10.07	5	10	16.88	4.10
R12	41.56	40	8.44	10	19.43	1.80
R13	70.9	10	9.1	10	8.45	3.04

**Table 6:** Analysis of variance (ANOVA) results for the quadratic model of the response bending stress (Y_1_).

Source	Sum of Squares	df	Mean Square	F-Value	*p*-Value
Model	280.18	5	56.04	253.96	<0.0001
Linear mixture	234.40	2	117.20	531.15	<0.0001
AB	45.51	1	45.51	206.24	<0.0001
AC	0.2184	1	0.2184	0.9896	0.3530
BC	0.0233	1	0.0233	0.1056	0.7548
Residual	1.54	7	0.2206		
Lack of fit	0.4579	3	0.1526	0.5618	0.6683
Pure error	1.09	4	0.2717		
Total	281.72	12			
R^2^	0.9945				
Adjusted R^2^	0.9906				
Predicted R^2^	0.9839				
Adequate precision	47.0840				

**Table 7:** Analysis of variance (ANOVA) results for the linear model of the response crystallinity index (Y_2_).

Source	Sum of Squares	df	Mean Square	F-Value	*p*-Value
Model	5.91	2	2.96	18.97	0.0004
Linear mixture	5.91	2	2.96	18.97	0.0004
Residual	1.56	10	0.1558		
Lack of fit	0.8471	6	0.1412	0.7948	0.6190
Pure error	0.7106	4	0.1776		
Total	7.47	12			
R^2^	0.7914				
Adjusted R^2^	0.7497				
Predicted R^2^	0.6243				
Adequate precision	12.0762				

**Table 8:** Criteria of variables and responses for the numerical optimisation process.

Variable	Goal	Lower Limit	Upper Limit	Importance
Eudragit E PO (% *w*/*w*)	Is in range	40	75	3
Kollidon 17 PF (% *w*/*w*)	Is in range	10	40	3
Stearic acid (% *w*/*w*)	Is in range	5	10	3
Bending stress (N/mm^2^)	Minimise	6.5	22.0	1
Crystallinity index	Minimise	1.8	4.1	5

**Table 9:** Results of the confirmation runs (*n* = 3)) used for model validation by comparison of the predicted and experimental values. (CI = Confidence Interval, Std. = Standard).

Response	95% CI Low	Predicted Mean	Experimental Mean	95% CI High	Predicted Std. Error	Std. Deviation
Bending Stress	11.2957	12.2199	12.9974	13.1441	0.400794	0.442695
Crystallinity Index	1.08774	1.79015	1.74145	2.49256	0.315247	0.394677

## Data Availability

Data will be shared upon request.
